# Correction: Mitochondrionopathy Phenotype in Doxorubicin-Treated Wistar Rats Depends on Treatment Protocol and Is Cardiac-Specific

**DOI:** 10.1371/journal.pone.0097795

**Published:** 2014-05-09

**Authors:** The PLOS ONE Staff

## Notice of Republication

This article was republished on April 24, 2014, to correct errors in in-text references and the missing Figure 4 in the PDF version of the article. Please download this article again to view the correct version. The originally published, uncorrected article and the republished, corrected article are provided here for reference.

## Supporting Information

File S1 Originally published, uncorrected article.
(PDF)

File S2 Republished corrected article.
(PDF)

## References

[1] Pereira GC, Pereira SP, Pereira CV, Lumini JA, Magalhães J, et al (2012) Mitochondrionopathy Phenotype in Doxorubicin-Treated Wistar Rats Depends on Treatment Protocol and Is Cardiac-Specific. PLoS ONE 7(6): e38867 doi:10.1371/journal.pone.0038867 22745682 10.1371/journal.pone.0038867 PMC3382146

