# Supplementary figures and images for: Mitochondrionopathy Phenotype in Doxorubicin-Treated Wistar Rats Depends on Treatment Protocol and Is Cardiac-Specific
Source: PLoS One. 2012 Jun 22;7(6):e38867. doi: 10.1371/journal.pone.0038867 (PMC3382146; doi:10.1371/journal.pone.0038867)

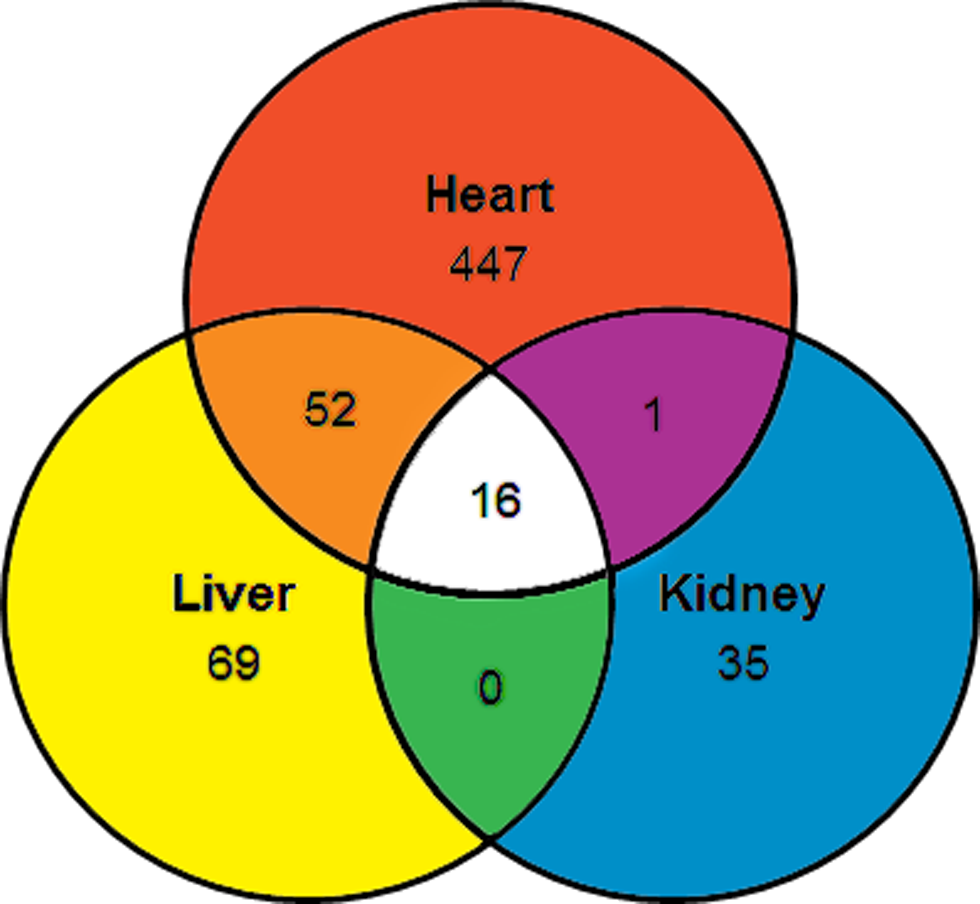

Supplement: Figure S1 — PubMed results distribution of research involving “doxorubicin” and “mitochondria” according to tissue category. The Venn diagram presented in the figure was elaborated after collecting data from the PubMed website (assessment date February 27th) using specific #keywords to obtain the desired output. Briefly, papers in the database that included works related to the #drug and #mitochondria were retrived, restricting the output for research performed in the defined #tissue, excluding #reviews and works performed in #humans as long as they are not indexed with other animals. Therefore, the base of the search string was as follow: (((#mitochondria AND #drug) AND #tissue) NOT #reviews) NOT #humans Further explanation about each of the keywords is given in supporting Table 1. The authors recognize that the present search string is not flawless; however, the idea is to give the reader an overview of report rankings across the selected tissues. In fact, we acknowledge the fact that, for example, the keyword #humans will not include recent reports since they are yet to be indexed to Medline. (TIF) [file pone.0038867.s001.tif]
