# Supplementary material for: Mitochondrionopathy Phenotype in Doxorubicin-Treated Wistar Rats Depends on Treatment Protocol and Is Cardiac-Specific
Source: PLoS One. 2012 Jun 22;7(6):e38867. doi: 10.1371/journal.pone.0038867 (PMC3382146; doi:10.1371/journal.pone.0038867)
Supplement: Table S1 — Description of keywords used in PubMed search for construction of Venn diagram of Fig. S1 , as well as the number of results retrieved with for each corresponding keyword. (DOCX) [file pone.0038867.s002.docx]

**Supplementary Table S1 – Description of keywords used in PubMed search for construction of Venn diagram of Fig.5, as well as the number of results retrieved with for each corresponding keyword.**

| No. | Keyword | Search terms | Objective | Search string | Number of results |
| --- | --- | --- | --- | --- | --- |
| 1 | #mitochondria | mitochondri* | truncation is used to retrieve all possible terms related to mitochondria, e.g. mitochondrion, mitochondrial, mitochondriac, mitochondrionopathy, etc. | -- | -- |
| 2 | #drug | (doxorubicin OR adriamycin) | sometimes reports use the term adriamycin rather than doxorubicin; allow inclusion of non-MeSH indexed reports | -- | -- |
| 3 | #reviews | (review[tiab] OR review[pt]) | exclude review publication even if they are not yet indexed by searching the word *review* in title or abstract of the report | -- | -- |
| 4 | #humans | (humans[mh] NOT animals[mh:noexp]) | exclude indexed reports performed in humans as long as they are not indexed with other animals | -- | -- |
| 5 | #tissue | (heart OR liver OR kidney) | reports which include any of the tissues | (((1 AND 2) AND 5) NOT 3) NOT 4 | 534 |
| 6 |  | heart | reports which include the selected tissue but might also include any of the other non-mentioned tissues or both | (((1 AND 2) AND 6) NOT 3) NOT 4 | 447 |
| 7 |  | liver |  | (((1 AND 2) AND 7) NOT 3) NOT 4 | 138 |
| 8 |  | kidney |  | (((1 AND 2) AND 8) NOT 3) NOT 4 | 35 |
| 9 |  | (heart AND liver NOT kidney) | reports which include the both selected tissues but exclude the last one | (((1 AND 2) AND 9) NOT 3) NOT 4 | 52 |
| 10 |  | (heart AND kidney NOT liver) |  | (((1 AND 2) AND 10) NOT 3) NOT 4 | 1 |
| 11 |  | (liver AND kidney NOT heart) |  | (((1 AND 2) AND 11) NOT 3) NOT 4 | 0 |
| 12 |  | (heart AND liver AND kidney) | reports which include all tissues together | (((1 AND 2) AND 12) NOT 3) NOT 4 | 16 |
| 13 |  | (heart NOT liver NOT kidney) | reports which include only the selected tissue | (((1 AND 2) AND 13) NOT 3) NOT 4 | 378 |
| 14 |  | (liver NOT heart NOT kidney) |  | (((1 AND 2) AND 14) NOT 3) NOT 4 | 69 |
| 15 |  | (kidney NOT heart NOT liver) |  | (((1 AND 2) AND 15) NOT 3) NOT 4 | 17 |
