# Supplementary material for: Correction: Mitochondrionopathy Phenotype in Doxorubicin-Treated Wistar Rats Depends on Treatment Protocol and Is Cardiac-Specific
Source: PLoS One. 2014 May 9;9(5):e97795. doi: 10.1371/journal.pone.0097795 (PMC4016360; doi:10.1371/journal.pone.0097795)
Supplement: File S2 — Republished corrected article. (PDF) [file pone.0097795.s002.pdf]

PLoS one

Mitochondrionopathy Phenotype in Doxorubicin-Treated Wistar Rats Depends on Treatment Protocol and Is Cardiac-Specific

Gonçalo C. Pereira[1], Susana P. Pereira[1], Claudia V. Pereira[1], José A. Lumini[2,3], José Magalhães[2], António Ascensão[2], Maria S. Santos[1], António J. Moreno[4], Paulo J. Oliveira[1]*

1 Department of Life Sciences, Center for Neuroscience and Cell Biology (CNC), University of Coimbra, Coimbra, Portugal, **2** Faculty of Sport Sciences, Research Centre in Physical Activity, Health and Leisure, University of Porto, Porto, Portugal, **3** Faculty of Health Sciences, University of Fernando Pessoa, Porto, Portugal, **4** Department of Life Sciences, Institute for Marine Research, University of Coimbra, Coimbra, Portugal

Abstract

Although doxorubicin (DOX) is a very effective antineoplastic agent, its clinical use is limited by a dose-dependent, persistent and cumulative cardiotoxicity, whose mechanism remains to be elucidated. Previous works in animal models have failed to use a multi-organ approach to demonstrate that DOX-associated toxicity is selective to the cardiac tissue. In this context, the present work aims to investigate *in vivo* DOX cardiac, hepatic and renal toxicity in the same animal model, with special relevance on alterations of mitochondrial bioenergetics. To this end, male Wistar rats were sub-chronically (7 wks, 2 mg/Kg) or acutely (20 mg/Kg) treated with DOX and sacrificed one week or 24 hours after the last injection, respectively. Alterations of mitochondrial bioenergetics showed treatment-dependent differences between tissues. No alterations were observed for cardiac mitochondria in the acute model but decreased ADP-stimulated respiration was detected in the sub-chronic treatment. In the acute treatment model, ADP-stimulated respiration was increased in liver and decreased in kidney mitochondria. Aconitase activity, a marker of oxidative stress, was decreased in renal mitochondria in the acute and in heart in the sub-chronic model. Interestingly, alterations of cardiac mitochondrial bioenergetics co-existed with an absence of echocardiograph, histopathological or ultra-structural alterations. Besides, no plasma markers of cardiac injury were found in any of the time points studied. The results confirm that alterations of mitochondrial function, which are more evident in the heart, are an early marker of DOX-induced toxicity, existing even in the absence of cardiac functional alterations.

Citation: Pereira GC, Pereira SP, Pereira CV, Lumini JA, Magalhães J, et al. (2012) Mitochondrionopathy Phenotype in Doxorubicin-Treated Wistar Rats Depends on Treatment Protocol and Is Cardiac-Specific. PLoS ONE 7(6): e38867. doi:10.1371/journal.pone.0038867

Editor: Valdur Saks, Université Joseph Fourier, France

Received March 2, 2012; **Accepted** May 13, 2012; **Published** June 22, 2012

Copyright: © 2012 Pereira et al. This is an open-access article distributed under the terms of the Creative Commons Attribution License, which permits unrestricted use, distribution, and reproduction in any medium, provided the original author and source are credited.

Funding: The present work was funded by the Foundation for Science and Technology (FCT), Portugal: research grants PTDC/SAU-OSM/64084/2006 and PTDC/SAU-OSM/104731/2008 (PJO), through FEDER/COMPETE/National funds; Post-Doc fellowships SFRH/BPD/42525/2007 to AA and SFRH/BPD/66935/2009 to JM; and, Ph.D. fellowships SFRH/BD/30906/2006 to JAL, SFRH/BD/36938/2007 to GCP, SFRH/BD/48029/2008 to CVP, SFRH/BD/64247/2009 to SPP. The funders had no role in study design, data collection and analysis, decision to publish, or preparation of the manuscript.

Competing Interests: The authors have declared that no competing interests exist.

* E-mail: pauloliv@ci.uc.pt

Introduction

Doxorubicin (DOX) is an anthracycline antibiotic drug which has been effectively and widely used in the clinic to treat several types of human and non-human cancers [1]. However, adverse side effects can be observed during or after treatment cessation, with a dose dependent and cumulative cardiotoxicity being the most complex and difficult to manage event [1,2]. In fact, the associated risk of developing congestive heart failure was one of the main reasons that lead to limitation of the maximum allowed dosage during DOX treatment [3].

Acute cardiac toxicity occurs early during the treatment and usually includes myopericardits, sinus tachycardia, reversible arrhythmias, prolonged QT interval and flattening of the T wave, being easily manageable and disappearing once the treatment is ceased [2]. Alternatively, patients may develop chronic cardiotoxicity which can appear right after the end of the treatment or even years later [2,4]. Unlike acute toxicity, the dose-dependence together with its difficult early detection makes chronic toxicity a life-threatening and largely uncontrolled condition.

The mechanisms underlying DOX-selective cardiotoxicity have been the focus of interest in the last four decades, but there is hardly any consensual conclusion. Nevertheless, it is accepted that DOX antitumor activity is completely independent from cardiac toxicity, which may involve disruption of mitochondrial function [2,5]. Distinctive features of DOX-induced mitochondrial dysfunction in the cardiac tissue include inhibition of oxidative phosphorylation, decreased calcium-loading capacity and increased reactive oxygen species (ROS) production [2,5,6].

Despite the fact that DOX toxicity has been described as being cardiac selective, none of the hypotheses proposed to date [2] fully explains the pronounced cardiac effects when compared with other vital tissues. Although several animal models have been generated to investigate either sub-chronic [7,8,9,10,11] or acute [12,13,14] DOX toxicity, one limitation of the majority of studies is that only one organ was investigated. Thus, comparing DOX multi-organ effects from different reports becomes non-accurate,

confusing and sometimes contradictory since animals species, strain, age and treatment protocols differ from publication to publication. By searching PubMed for reports using the keywords "mitochondria" and "doxorubicin" and narrowing the search to works performed in animal models and in three distinct tissues (heart, liver and kidney, Table S1), only 16 hits were obtained (Fig. S1). These studies investigated DOX toxicity in the three tissues harvested from the same experimental protocol. However, except for two relatively recent works, most reports originated in the 80 s and did not compare mitochondrial dysfunction with pathophysiological state, genetics, metabolomics or proteomics, which we believe are critical to better understand DOX-induced tissue toxicity. In an attempt to increase the knowledge regarding DOX-induced selective cardiotoxicity, the objective of the present work was to measure alterations of mitochondrial bioenergetics in the heart, liver and kidney after two distinct treatment protocols (acute vs. sub-chronic) in male Wistar rats. Mitochondrial function end-points were associated with tissue histological alterations (all three tissues) and function (heart). Our hypothesis is that alterations of mitochondrial bioenergetics occur predominantly in the heart and are an early and sensitive marker of DOX-induced toxicity, occurring even in the absence of histological alterations. A second tandem hypothesis is that cardiac mitochondrial toxicity is detectable only in the sub-chronic treatment.

The two distinct treatment protocols used in the present work were already reported in the literature [13,15] and are accepted models to investigate DOX-mitochondrionopathy. Note that accordingly to a reported equation [14], the actual total dosages of our treatment protocols are slightly below the maximum dosage allowed in human chemotherapy [16], as our objective was not to induce substantial tissue damage but to mimic biochemical and functional alterations that are usually observed in DOX-treated patients.

Materials and Methods

Reagents

DOX hydrochloride, (7S,9S)-7-[(2R,4S,5S,6S)-4-amino-5-hydroxy-6-methyloxan-2-yl]oxy-6,9,11-trihydroxy-9-(2-hydroxyacetyl)-4-methoxy-8,10-dihydro-7H-tetracene-5,12-dione hydrochloride, chemical purity ≥98%, was obtained from Sigma-Aldrich Quimica SA (Sintra, Portugal) and prepared in a sterile saline solution, NaCl 0.9% (pH 3.0, HCl) and stored at 4°C for no longer than five days upon rehydration. All other chemicals were of the highest grade of purity commercially available. Aqueous solutions were prepared in ultrapure (type I) water (Milli-Q Biocel A10 with pre-treatment via Elix 5, Millipore, Billerica, MA, USA). For non-aqueous solutions, ethanol (99.5%, Sigma-Aldrich Quimica SA, Sintra, Portugal) was used as solvent.

Animal Care

Animal handling was performed in accordance with the European Convention for the Protection of Vertebrate Animals used for Experimental and Other Scientific Purposes (CETS no.123) and Portuguese rules (DL 129/92). The procedures were approved by the CNC Committee for Animal Welfare and Protection. Animal handlers and the authors GCP, SPP and PJO are credited by the European Federation for Laboratory Animal Research (FELASA) category C for animal experimentation (accreditation no. 020/08). Fourteen weeks of age (acute protocol) or 6 weeks of age (sub-chronic protocol) male Wistar rats, Crl:WI(Han), were purchased from Charles River (France), acclimated for 10–14 days prior to the initiation of experiments and maintained in the local animal house facility (CNC – School of Medicine, University of Coimbra, Coimbra, Portugal). Animals were group-housed in type III-H cages (Tecniplast, Italy) with irradiated corn cob grit bedding (Scobis Due, Mucedola, Italy) and environmental enrichment and under controlled environmental requirements (22°C, 45–65% humidity, 15–20 air changes/hour, 12 h artificial light/dark cycle, noise level <55 dB) and free access to standard rodent food (4RF21 GLP certificate, Mucedola, Italy) and *ad libitum* acidified water (at pH 2.6 with HCl).

Experimental Design

For the acute study, the experimental protocol was initiated with 16 weeks old rats (N = 34), randomly allocated in pairs and administered with either DOX (20 mg Kg^{-1} of body weight, i.p., n = 17) or with an equivalent volume of vehicle solution (NaCl 0.9%, i.p., n = 17) exactly 24 hours before sacrifice, as previously described [13].

For the sub-chronic study, the experimental protocol was performed with 8 weeks old rats (n = 40) randomly grouped in pairs and weekly injected with a subcutaneous injection in the scruff or flank of either DOX (2 mg kg^{-1}, n = 20) or equivalent volume of vehicle solution (NaCl 0.9%, s.c., n = 20) during seven weeks, and sacrificed one week after the last injection, as previously described [15].

All animals were injected during the light phase of the cycle, observed daily and weighed at the beginning and at the end of the experimental treatment period, being also weekly weighed at the time of injection. Animals were euthanized in pairs by cervical dislocation followed by decapitation, to confirm death and exsanguination. Blood was collected for further biochemical analysis. Rats were sacrificed between 9:00 and 10:00 AM to eliminate possible effects due to diurnal variation and were not fasted before sacrifice. Organs were immediately extracted from the body and quickly washed in appropriate buffer before being weighed.

Blood Analysis

Blood was collected after decapitation to sterile tubes without additives. After blood clot formation, serum was separated by centrifugation at 1,600×g during 10 minutes at 4°C (Sigma 3–16 K, 1333 rotor). The supernatant was then transferred to microtubes and centrifuged at 16,000×g, 20 minutes at 4°C (Eppendorf 5415 R, FL062 rotor). Serum samples were maintained for a short time at 4°C for analysis by an external certified laboratory, or stored frozen at −80°C for troponin I (TnI) analysis. The latter was performed by external trained personal one month after collection using the singleplex Rat Cardiovascular Panel (RCVD1-89K, Millipore, Arium, Portugal). Blood analyses were performed by blinded operator.

Histological Analysis

Organs were immersion-fixed in Bouin's solution for 24 h and then washed with 70% alcohol until the solution became clear and stored in 70% alcohol until the histological analysis was performed. At that time, after several incubations with increasing alcohol percentages (70%, 90%, and 100%) and xylol, tissues were processed using a normal paraffin procedure and sectioned (3 µm thick). The sections were then de-paraffinized with xylol and incubated with solutions containing decreasing alcohol content (100% and 95%). All slides were stained with hematoxilin and eosin (HE) by using standard procedures. The samples were covered with coverslips in Eukitt mounting medium and then visualized in a Nikon Eclipse 80I microscope coupled with a camera and computer. Morphological assessment was conducted in a "blind" fashion by a certified professional.

Electron Microscopy

After organ harvesting and washing, a small slice (2–5 mm) was cut and fixed in 3% glutaraldehyde in phosphate buffer (100 mM NaH_2PO_4, pH 7.3), postfixed in 1% osmium tetroxide in same buffer and dehydrated in solutions containing increasing alcohol percentages (70%, 90%, and 100%) before being embedded in Spurr's resin. Ultrathin sections were obtained with an LKB ultra-microtome Ultrotome III (GE Healthcare, Little Chalfont, Buckinghamshire, UK), stained with methanolic uranyl acetate followed by lead citrate, and examined with a JEOL Jem-100SX electron microscope (JEOL, Tokyo, Japan) operated at 80 kV. The operator was blinded to treatment groups and took 5 to 10 micrographs of random fields.

Echocardiogram

Echocardiograms were performed under the same specifications as previously described [17]. Five days after the last injection, sub-chronically-treated animals, free of anesthesia, were examined lying in the left lateral decubitus position and using a commercial available echocardiograph system (VIVID i, G.E. Helthcare), equipped with an 11.5 MHz transducer. Every parameter was measured accordingly to the American Society for Echocardiography guidelines (www.asecho.org/guidelines/) and results were directly obtained from the equipment software by a cardiologist blinded for treatment groups. Left ventricular mass (LV mass) was calculated using a standard cube formula which assumes a spherical left ventricular geometry [18] according to the following equation: $LVmass = 1.04 \times [(LVDd + LVPW + IVS)^3 - LVDd^3]$. Results are expressed in mg and 1.04 represents the specific gravity of muscle. Only four animals of each group were analyzed due to limitations in the time slot available for the cardiologist/apparatus used.

Isolation of Mitochondrial Fractions

Mitochondria were isolated by the standard procedure usually used in our laboratory [19,20,21]. Organs were excised and finely minced in an ice-cold isolation medium containing 250 mM sucrose, 10 mM HEPES, 1 mM EGTA and 0.1% defatted BSA (pH 7.4, KOH). For the isolation of cardiac mitochondrial fractions, the isolation medium was supplemented with 0.5 µg/mL of protease (Subtilisin A, Type VIII from *Bacillus licheniformis*, Sigma-Aldrich). The mitochondrial protein after isolation was quantified by the biuret method using bovine serum albumin as a standard [22] and mitochondrial preparation was kept on ice during experiments, which were carried out after a 20 min recovery and within 5 hours post-isolation.

Oxygen Consumption

Oxygen consumption of isolated mitochondria was monitored polarographically with a Clark oxygen electrode connected to a Kipp and Zonen recorder in a 1 mL thermostatic, water-jacketed open chamber with magnetic stirring at 30°C, simultaneously with mitochondrial membrane potential measurements (see 2.10). The standard respiratory medium consisted of 130 mM sucrose, 10 µM EGTA, 50 mM KCl, 5 mM H_2PO_4, 5 mM HEPES (pH 7.3, KOH) and supplemented with 2.5 mM $MgCl_2$ for liver and kidney. Mitochondria were suspended at a concentration of 0.5 (heart) or 1.0 (liver and kidney) mg protein/mL in the respiratory medium and mitochondrial state 2 respiration was initiated with 5 mM glutamate plus malate (mitochondrial energization through complex I) or 5 mM succinate in the presence of 2 µM rotenone (mitochondrial energization through complex II). Adenosine diphosphate (ADP) (225–250 nmol) was added to initiate state 3 respiration. State 4 respiration was defined as oxygen consumption after ADP consumption. The respiratory control ratio (RCR) was calculated as the ratio between state 3 over state 4 and it is an indicator of oxidative phosphorylation coupling and mitochondrial membrane integrity. ADP/O ratio, which is expressed as the ratio between the amount of ADP added and oxygen consumed during state 3 respiration, is an index of oxidative phosphorylation efficiency. Respiration rates were calculated considering that the saturation oxygen concentration was 236 µM at 30°C.

Mitochondrial Transmembrane Electric Potential

The mitochondrial transmembrane electric potential ($\Delta\Psi_{max}$) was monitored by tetraphenylphosphonium ion (TPP^+) distribution (see 2.9), by using a TPP-selective electrode in combination with a Ag/AgCl saturated reference electrode, as previously described [21]. The difference in potential between the selective TPP^+ electrode and the reference electrode was measured with a potentiometer and continuously recorded in a Kipp and Zonen recorder (model BD 121; Kipp & Zonen B.V., Delft, Netherlands). Experimental conditions were the same as for oxygen consumption assays with the inclusion of 3 µM TPP^+ in the reaction media. Absolute $\Delta\Psi_{max}$ values (mV) were determined from the equation originally proposed by Kamo [23], assuming Nernst distribution of the ion across the membrane electrode. No correction was made for the "passive" binding of TPP^+ to mitochondrial membranes because the purpose of the experiments was to show relative changes in potential rather than absolute values. As a consequence, some overestimation for the $\Delta\Psi_{max}$ values may be anticipated. A matrix volume of 1.1 µL/mg protein was assumed.

Aconitase Activity

Mitochondrial protein (200 µg) was diluted in 0.6 ml buffer containing 50 mM Tris–HCl (pH 7.4) and 0.6 mM $MnCl_2$ and sonicated for 10–20 seconds, followed by centrifugation at 16,000×g for 5 minutes. Aconitase activity was immediately spectrophotometrically measured (Jasco V-560, Jasco Europe, Milan, Italy) by monitoring the formation of *cis*-aconitate from isocitrate at 240 nm in 50 mM Tris-HCl (pH 7.4) containing 0.6 mM $MnCl_2$ and 20 mM isocitrate at 25°C [24]. All assays were performed in triplicate. Enzyme activity was calculated by using the mean of the slopes of the three replicates, obtained before the record reached a plateau. Results were expressed as percentage of control, which was 174.0 ± 27.8 nmol $mg_{protein}^{-1}$ - min^{-1}, 258.3 ± 32.4 nmol $mg_{protein}^{-1}$ min^{-1} and 245.2 ± 31.1 nmol $mg_{protein}^{-1}$ min^{-1} for heart, liver and kidney, respectively, using $\varepsilon_{240} = 3.6$ mM^{-1} cm^{-1} [24].

Statistical Analysis

All data was assessed for normality using the Kolmogorov-Smirnov test with Dallal-Wilkinson-Lilliefor correction and for equality of variances using the F test. Since group sample sizes are equal and the parametric statistical tests applied in this work are robust for moderate deviations from homoscedasticity [25], parametric tests were still applied when homoscedasticity was not observed. However, when data normality was rejected, a squared-root, logarithmic or reciprocal transformation was applied in an attempt to achieve normality. If data still rejected normality, the correspondent non-parametric statistical test was used. Nevertheless, data is presented to the reader in non-transformed values for ease of comprehension. In the text, data is expressed as percentage of the difference of means plus its standard error or as percentage of the means difference plus its standard error for data related to isolated mitochondrial fractions. In both situations, the

percentage value shown regards the saline group in the same experimental model. Statistical significance between means was determined using two-tailed Student's t test. When homoscedasticity was not found, Welch's correction was applied and in the absence of normality, the Mann-Whitney test was used instead. To exclude the random effect associated with daily mitochondrial isolation and electrode variability, a matched pairs Student's t test or its non-parametric correspondent Wilcoxon matched pairs test were performed. Differences were considered significant at 5% level and p value was categorized accordingly to their interval of confidence. Statistical analyses were performed using Graph Pad Prism version 5.0 (GraphPad Software, Inc., San Diego, CA, USA).

Results

Animal and Organs Mass

Control and DOX-treated animals in the acute model did not show alterations in their body mass in the 24 hours subsequent to DOX administration (Table 1). However, when animals were individually analyzed, i.e. when the mean of the arithmetic difference between initial and final body mass of every individual was calculated for the first 24 h, the control group showed a body mass variation of -0.35 ± 1.02 g (n = 17) while variation in DOX-treated group, -15.1 g ±1.7 g (n = 17) representing about 4% of total body mass, was significantly lower ($p<0.001$). Surprisingly, a decrease of 7.6±2.7% in heart mass was observed 24 h after the single DOX injection but no alteration was observed in other tissues analyzed (1.6±4.4% increase in liver and 3.1±3.9% decrease for kidney, Table 1). Therefore, heart mass over body mass ratio showed a significant decrease of 5.8±2.3% while no changes were found for liver or kidney (3.6±2.6% increase and 1.4±3.6% decrease, respectively; Table 1).

Treatment of Wistar rats with seven weekly DOX injections caused a significant reduction in body mass gain of animals, as seen by the difference of body mass values between groups (13.8±2.1%; Table 1). While the body mass variation of the control group during the treatment showed an increase of 49.1±2.9%, the DOX-treated group only increased body mass by 31.2±3.1%, which can be easily observed in the body mass gain profile depicted in Fig. 1. However, it was interesting to observe that the alteration in heart mass detected in the acute model was not present in the sub-chronic protocol at the end of the treatment period (Table 1). Since only a non-significant decrease of all tissues (2.4±5.6%, 3.2±4.0% and 5.2±3.6% for heart, liver and kidney, respectively) was observed, the ratio of organ mass to body mass was increased in all analyzed tissues (13.7±6.6%, 12.4±3.5% and 10.0±4.1% for heart, liver and kidney, respectively; Table 1). The results reflect a change in body mass rather than alterations in tissue mass.

It should be noticed that although the objective of the experimental protocol was to induce a sub-chronic response and therefore a lower rate of mortality, one animal died during DOX treatment (marked with an arrow, Fig. 1). This treated animal received all seven DOX injections and died precisely one week after the last. Nonetheless, the animal did not show any distinct sign of distress or illness when compared with its counterparts. A standard necropsy was performed in the deceased animal but no particular abnormality was perceived, including heart hypertrophy or ascites. Liver and kidney tissues appeared normal in size, morphology and color. The only thing to point out was the fact that this particular animal showed the larger decrease in weight. Therefore, the mortality rate associated with the present study is 5% (1 out of 20).

Table 1. Body and organs mass profile of animals subjected to DOX treatment protocols.

Model	Treatment	Initial body mass (g)		Final body mass (g)		Heart mass (g)		HM:BM (×1000)		Liver mass (g)		LM:BM (×1000)		Kidney mass (g)		KM:BM (×1000)	
		Mean	SE	Mean	SE	Mean	SE	Mean	SE	Mean	SE	Mean	SE	Mean	SE	Mean	SE
Acute	Saline (n = 17)	377.5	7.8	377.2	7.9	0.972	0.020	2.58	0.05	11.6	0.3	30.8	0.05	2.19	0.05	5.84	0.02
	DOX (n = 17)	385.1	9.7	370.0	9.6	0.898**	0.018	2.44*	0.04	11.8	0.4	32.9	0.07	2.13	0.07	5.76	0.02
Sub-Chronic	Saline (n = 19)	263.0	5.0	391.8	5.9	1.25	0.04	3.20	1.29	12.3	0.4	31.4	0.07	2.50	0.06	6.40	0.02
	DOX (n = 19)	255.4	5.3	337.4***	5.8	1.22	0.05	3.64*	1.65	11.9	0.3	35.3**	0.08	2.36	0.06	7.03*	0.02

Data refers to wet organ mass and its ratio to body mass was obtained dividing the organ mass over the respective total animal mass times 1000. The deceased DOX-treated rat and its matched control in the sub-chronic model were excluded from this analysis. Differences between treatment groups means within the same model were evaluated by Student's t test (see Material and Methods for detailed information).
*p≤0.05;
**p≤0.01;
***p≤0.001 vs saline group of the same treatment protocol. HM:BM – heart mass to body mass ratio; LM:BM – liver mass to body mass ratio; KM:BM – kidney mass to body mass ratio; SE – standard error.
doi:10.1371/journal.pone.0038867.t001

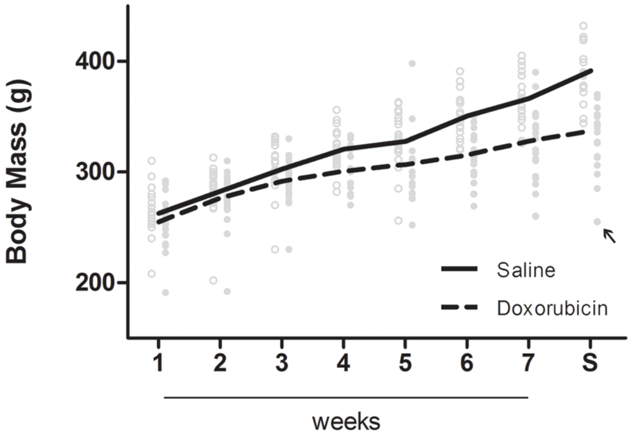

Figure 1. DOX decreases body mass gain over time in a sub-chronic toxicity model. After the fourth injection, the body mass of DOX-treated animals started to be distinctly different from the saline-treated and therefore the growth profile is dramatically changed at the end of treatment. Only one DOX-treated animal died during the protocol (indicated by the black arrow). Animals in the control group are depicted by open circles while DOX-treated animals are in full circles. Lines represent the means of each group at each time point. S – sacrifice time-point.
doi:10.1371/journal.pone.0038867.g001

Serum Biochemistry

Despite the fact that lactate dehydrogenase (LDH), a general marker for tissue damage, is not altered (21.8±14.0%; Table 2) in the acute model, the non-specific hepatic marker, aspartate aminotransferase (AST), is markedly elevated 91.0±17.2%. Serum creatine kinase is unchanged (1.7±16.0%) as well as markers of renal function, namely urea, creatinine, uric acid or blood urea nitrogen (BUN). The observed high levels of the hepatic-specific marker, alanine aminotransferase (ALT), 141±26%, together with the lower AST to ALT ratio (15.3±7.5%) and total protein levels (TP; 9.8±1.7%) suggest impaired liver function in DOX-treated animals. Increased cardiac injury, as evaluated by measuring troponin I (TnI) was not significantly increased in the acute model (value increased 15±11% vs. control). Regarding circulating-blood lipids, triglycerides were decreased by 53±13% 24 hours after the acute treatment but no alteration was found in total cholesterol (4.6±7%).

Notwithstanding, chronically-DOX-treated rats were hyperlipidemic with the treated group showing both an increase in triglycerides and cholesterol (196±40% and 127±24%, respectively; Table 2). TnI levels were also increased by 11±12%, although the difference was not statistically significant. Interestingly, all other parameters analyzed were significantly decreased in comparison to control animals (average of the parameters variation of 17.3%, ranging from 8.7% to 31.4%), with the exception of LDH, uric acid and transaminases ratio, which, despite being also decreased in the DOX-treated group, did not reach statistical significance when compared with the saline group.

Echocardiography

Animals from sub-chronic DOX treatment were submitted to an echocardiogram 5 days after the 7th injection in order to evaluate cardiac morphology and function parameters. No abnormality was found in the four animals analyzed (Table 3). Thickness of the walls and left ventricular diameter, ejection fraction and fraction shortening were not different from the

Table 2. Blood plasma profile after DOX treatment.

Model	Treatment	CK (U/L)	TnI # (ug/L)	TRIG (mg/dL)	CHOL (mg/dL)	AST (U/L)	ALT (U/L)	AST/ALT	TP (g/dL)	LDH (U/L)	CREA (mg/dL)	Urea (mg/dL)	UA (mg/dL)	BUN (mg/dL)
Acute	Saline (n = 15)	13039±1555)	915.9±61.5)	114.5±14.3)	38.8±2.0)	178.7±15.3)	57.1±3.0)	3.11±0.18)	71.4±0.9)	2507±224)	0.59±0.02)	35.0±1.8)	1.71±0.14)	16.3±0.9)
	DOX (n = 15)	12816±1564)	1053.0±83.7)	54.1*** ±4.2)	40.6±1.9)	341.0*** ±26.9)	137.8*** ±14.6)	2.64* ±0.15)	64.4*** ±0.9)	3055±271)	0.57±0.02)	34.5±1.3)	1.66±0.09)	16.1±0.6)
Sub-Chronic	Saline (n = 18)	9130±1007)	840.3±57.4)	126.9±8.9)	41.0±1.2)	183.1±10.6)	51.5±2.5)	3.63±0.20)	71.5±0.6)	4224±432)	0.64±0.01)	38.8±0.7)	1.63±0.12)	18.1±0.3)
	DOX (n = 18)	6262* ±709)	932.6±86.1)	376.3*** ±49.6)	93.1*** ±9.7)	139.6** ±5.0)	43.9* ±1.5)	3.20±0.10)	62.3*** ±0.9)	3561±497)	0.58** ±0.01)	33.7*** ±0.7)	1.39±0.09)	15.7*** ±0.3)

#For troponin I analysis, only 13 and 16 samples were measured in acute and sub-chronic protocol, respectively due to limitations in the analytical kit used.
Differences between treatment groups means within the same model were evaluated by Student's t test, when assumptions were not achieved a Welch correction or the non-parametric Mann-Whitney test were applied (see Material and Methods for detailed information).
$*p \leq 0.05$;
$**p \leq 0.01$;
$***p \leq 0.001$ vs saline group of the same model. List of abbreviations: CK – creatine kinase; TnI – troponin I; TRIG – triglycerides; CHOL – total cholesterol; AST – aspartate aminotransferase; ALT – alanine aminotransferase; TP – total serum proteins; LDH – lactate dehydrogenase; CREA – creatinine; UA – uric acid; BUN – blood urea nitrogen; SE – standard error.
doi:10.1371/journal.pone.0038867.t002

Table 3. Echocardiogram parameters in the sub-chronic protocol.

		IVS (mm)		LPWT (mm)		LVDd (mm)		LVDs (mm)		LV mass (mg)		LVEF (%)		FS (mm)		AT s/d (bpm)	
**Model**	**Treatment**	**Mean**	**SE**	**Mean**	**SE**	**Mean**	**SE**	**Mean**	**SE**	**Mean**	**SE**	**Mean**	**SE**	**Mean**	**SE**	**Mean**	**SE**
Sub-Chronic	Saline (n = 4)	1.49	0.05	1.54	0.05	5.02	0.21	2.44	0.13	416.3	44.7	87.3	0.8	51.2	1.1	520.8	15.1
	DOX (n = 4)	1.50	0.03	1.55	0.02	5.00	0.22	2.45	0.16	411.5	23.3	87.0	1.2	51.2	1.3	538.3	16.0

Differences between treatment groups were evaluated by non-parametric Mann-Whitney test due to their lack of normality (see Material and Methods for detailed information). IVS – interventricular septum; LPWT – left posterior wall thickness; LVDd – left ventricular diastolic dimension; LVDs – left ventricular systolic dimension; LVEF – left ventricular ejection fraction; FS – fraction shortening; AT s/d – arterial tension systole/diastole.
doi:10.1371/journal.pone.0038867.t003

control group at this time point (average of the parameters variation of 0.25%, ranging from 0.16 to 0.40%).

Histopathology and Ultrastructure Analyses

When analyzing at least three different samples from each tissue and from each treatment protocol, no obvious sign of damage or morphological alterations were found after histological analysis (Fig. 2). Hearts from treated animals in both treatment protocols did not present signs of fibrosis or any hallmarks of later stages of DOX toxicity. However, in the representative pictures of cardiac slices, minor cytoplasmic vacuolization in the acute model (Fig. 2, Panels A and B) and also minor increase in cellular volume in the sub-chronic model (Fig. 2, Panels C and D) can be observed. Livers were morphologically normal; however, minor centrilobular dilation was observed, while hepatocytes showed citoplasmic heterogeneity due to vacuolization. Nevertheless, the vacuolization was more prominent in slices from the sub-chronic model (Fig. 2, Panels E–H). When renal slices were observed with hematoxylin eosin (HE) stain, no differences were found between control and treated group regardless of the treatment protocol used (Fig. 2, Panels I–L).

In terms of tissue ultrastructure, electron micrographs of cardiac slices from acutely treated animals (Fig. 3, Panel B and D) showed a cellular structure not dissimilar to control (Fig. 3, Panel A and C). The myofibrillar disorganization, cytoplasm vacuolization and swollen mitochondria usually observed after DOX treatment in other rodent models [26,27] were not present in the acute treatment model (Fig. 3, Panels A–D). In fact, myofibrillar Z-bands were well defined and with narrow A-bands (Fig. 3, Panels C and D). Likewise, kidney electron micrographs were similar to control counterparts (Fig. 3, Panels H–K). However, hepatic slices presented more heterogeneous cytoplasm with high numbers of vacuoles and lipid-like droplets (Fig. 3, Panel E). Moreover, liver mitochondria from DOX acute treated animals appear to be preferentially in the condensed conformation (Fig. 3, Panel G) rather than the orthodox one observed in control micrographs (Fig. 3, Panels D and F).

Regarding the sub-chronic model (Fig. 3, Panels L-X), cardiac samples from treated animals also showed normal sarcomeres with well-defined Z-bands and organized myofibrils (Fig. 3, Panels A–D). Nevertheless, the cytoplasm appeared to present more vacuolization (Fig. 3, Panel M) although mitochondrial morphology was not dissimilar from control micrographs (Fig. 3, Panels L and N). In liver tissue, the striking evidence is the abundance of small vacuoles in the cytoplasm of hepatocytes from sub-chronically treated animals (Fig. 3, Panel S) and, although mitochondria appear to be greater in volume in some images, the overall observation is that they are not different in morphology when compared with saline treatments (Fig. 3, Panels P and R). Mitochondria from renal slices appear in an intermediate conformation between orthodox and condensed forms (Fig. 3, Panel X).

Mitochondrial Bioenergetics

Mitochondrial bioenergetics was evaluated in the three tissues to detect distinctive DOX-induced alterations. State 3 respiration mimics an increase in workload which is observed *in vitro* after ADP-induced simulation. State 4 respiration is observed after all ADP is phosphorylated, representing a steady-state of the respiratory chain, controlled mostly by the passive and unspecific diffusion of protons through the inner mitochondrial membrane. Similarly, the phosphorylation lag phase represents the time needed for the phosphorylative system to convert all added ADP to ATP, i.e., the time elapsed to mitochondrial repolarization after ADP depolarization.

In the acute model, complex I-sustained mitochondrial state 3 respiration was increased in hepatic, decreased in renal and not altered in cardiac fractions (14.7±5.5%, 5.3±2.0% and 4.4±5.3%, respectively; Fig. 4). However, despite equal variation patterns observed for the lag phase, ranging from 13 to 17% between tissues, there was no statistical difference between groups (Table 4). The previous detected differences for complex I-sustained mitochondrial state 3 respiration were absent when substrates for complex II were used (4.1±6.0%, 5.9±7.9% and 6.7±6.7% for heart, liver and kidney, respectively). State 4 respiration, RCR and ADP/O remained unchanged in all fractions regardless of the respiration substrate used (Figs. 4 and 5). The same was observed for all other parameters related to mitochondrial membrane potential.

Cardiac mitochondria from sub-chronic DOX-treated animals presented decreased state 3 respiration when using both respiratory complexes (15.8±4.9% and 12.8±3.6% for complex I and II, respectively; Fig. 4) and lower state 4 respiration, although only statistically significant when substrates for complex II were used (12.0±6.6% and 19.8±4.2% for complex I and complex II, respectively). Likewise and in a complementary manner, lag phase was increased for both complex I- and complex II-sustained respiration (10.7±6.0% and 13.1±5.5%, respectively; Table 4) and $\Delta\Psi_{max}$ was only slight, yet significantly, decreased when substrates for complex II were used (1.1±0.5% and 2.2±0.6% for complex I and II, respectively).

Hepatic and renal mitochondrial fractions behaved similarly, both having decreased $\Delta\Psi_{max}$ with complex I substrates (1.9±0.5% and 1.6±0.4%, respectively; Table 4). Slower state 3 respiration (6.6±3.8%) in hepatic fractions and kidney (12.6±4.2%) with complex I substrates was also measured. Along with a decreased state 3 respiration, liver mitochondria also showed a longer phosphorylative lag phase (27.1±11.6%). RCR

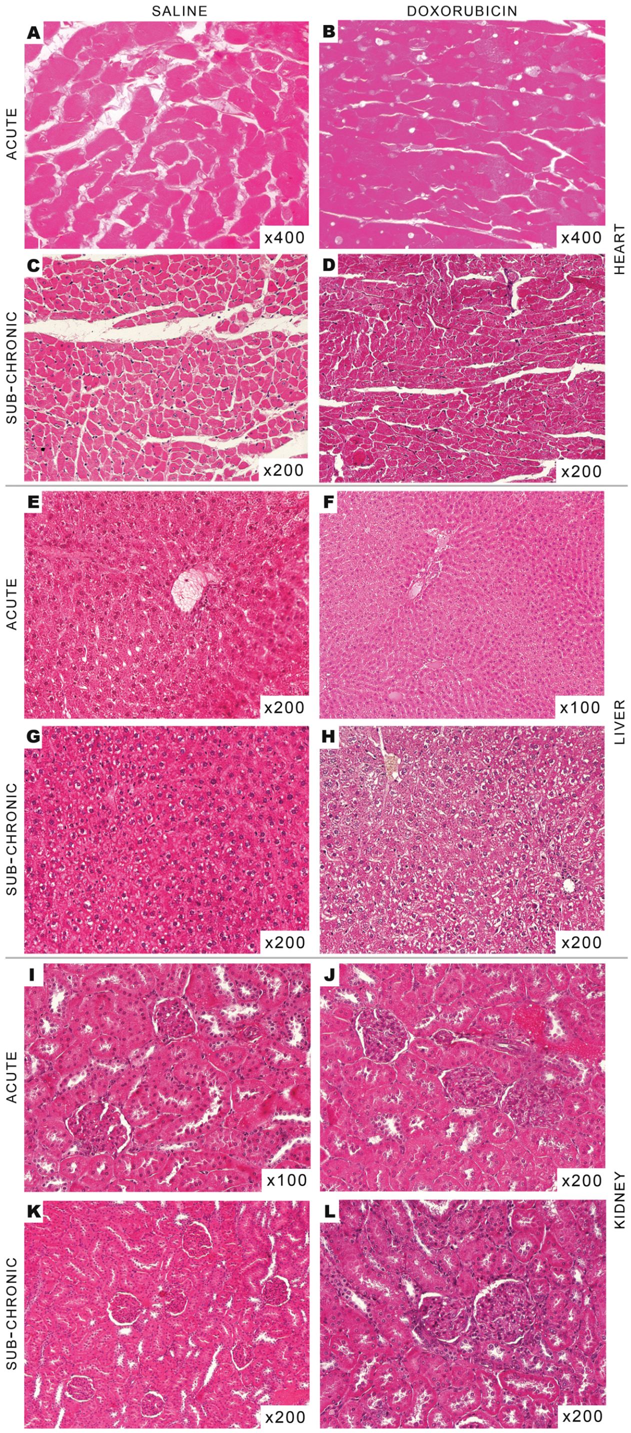

Figure 2. Histological analysis of organs collected from rats treated with DOX. No notorious differences or hallmarks of DOX toxicity were found in the different tissues in both protocols. Panels represent HE photographs of random chosen tissues: hearts present minor cytoplasmatic vacuolization (Panel **B**) and cytoplasmatic dilatation (Panel **D**); liver usually show minor cytoplasmatic vacuolization (Panel **F** and **H**); no changes in kidneys (Panel **J** and **L**). Organs were fixed in Bouin's solution, processed through standard histological procedures and stained with HE (for more information, see Material and Methods).

doi:10.1371/journal.pone.0038867.g002

and ADP/O values were similar between control and treated group in liver and renal mitochondrial.

Aconitase Activity

Aconitase activity was measured in all mitochondrial fractions as a marker of oxidative stress [24]. Alterations in aconitase activity were tissue-specific and treatment-dependent. The only tissue that showed a decreased enzyme activity in the acute model was the kidney (27.8±6.7%) while the activity remained unaltered in the heart and liver (0.3±11.8% and 15±12.3%, respectively; Table 5). Cardiac mitochondrial aconitase in the chronic model was decreased by 21.5±7.7% in the DOX-treated group while activity in the two other organs were unaltered (12.9±15.6% and 5.4±9.0% for liver and kidney, respectively).

Discussion

The present work demonstrates that DOX treatment induced different responses depending on the schedule protocol used. Transaminases and total serum protein levels suggest that the acute treatment affects the liver. Minor cytoplasmic vacuolization observed in histological and electron microscopy of thin slices from hepatic tissue may also suggest metabolic alterations in hepatocytes, which are supported by decreased triglyceride levels, despite no alterations in plasma cholesterol (Table 2). Moreover, the slight increase of state 3 respiration in the presence of complex I-linked substrates (Fig. 4), which are the most important in a cellular context, and adoption of the condensed mitochondrial conformation as seen by electron microscopy (Fig. 3), support the idea that hepatocyte metabolism and viability are affected after the acute treatment. Nevertheless, it is however unclear at the moment if alterations in lipid metabolism can contribute to worsen the cardiovascular fitness in treated animals. However, hyperlipidemia was clearly observable in the sub-chronic treatment and liver histology showed slightly more vacuolization that in the acute model (Table 2 and Fig. 2), suggesting that altered lipid metabolism may be a secondary response to drug treatment.

It is intriguing that sub-chronically-treated animals showed only increase in plasma lipids, while other parameters and markers were consistently decreased even those which are usually increased in chronic exposure to DOX [16,28,29] (Table 2). Nevertheless, because no substantial alterations in histology and ultrastructure analysis were also detected (Fig. 2 and 3), we believe that the organism of treated animals reached a new adaptive steady-state following sub-chronic DOX toxicity. Nevertheless, organ alterations may probably exist undetected which may lead to a disrupted response when subjected to metabolic or physiological stress.

Another interesting difference between treatments relates to heart mass which was the only organ in both models to show an alteration in this parameter. However, it was surprising to observe a difference only in the acute model since hypertrophy is usually reported along with DOX-induced cardiotoxicity [1,2,30]. Also surprising was the 7% decrease in heart mass after 24 h of treatment (Table 1). One hypothesis relates to apoptotic and/or necrotic events associated with DOX peak dosage in the plasma [29] and often observed in cardiac cells exposed to DOX [31]. Nevertheless, a previous work showed that a single injection of 10 mg/Kg DOX caused primarily a decrease in heart mass followed by a restoration to control values [32], which may explain why no alterations were observed in the sub-chronic model. However, sample size in the mentioned study was too small for a good interpretation of results. Nevertheless the authors explained the weight recovery as an increase in cytoplasm volume and

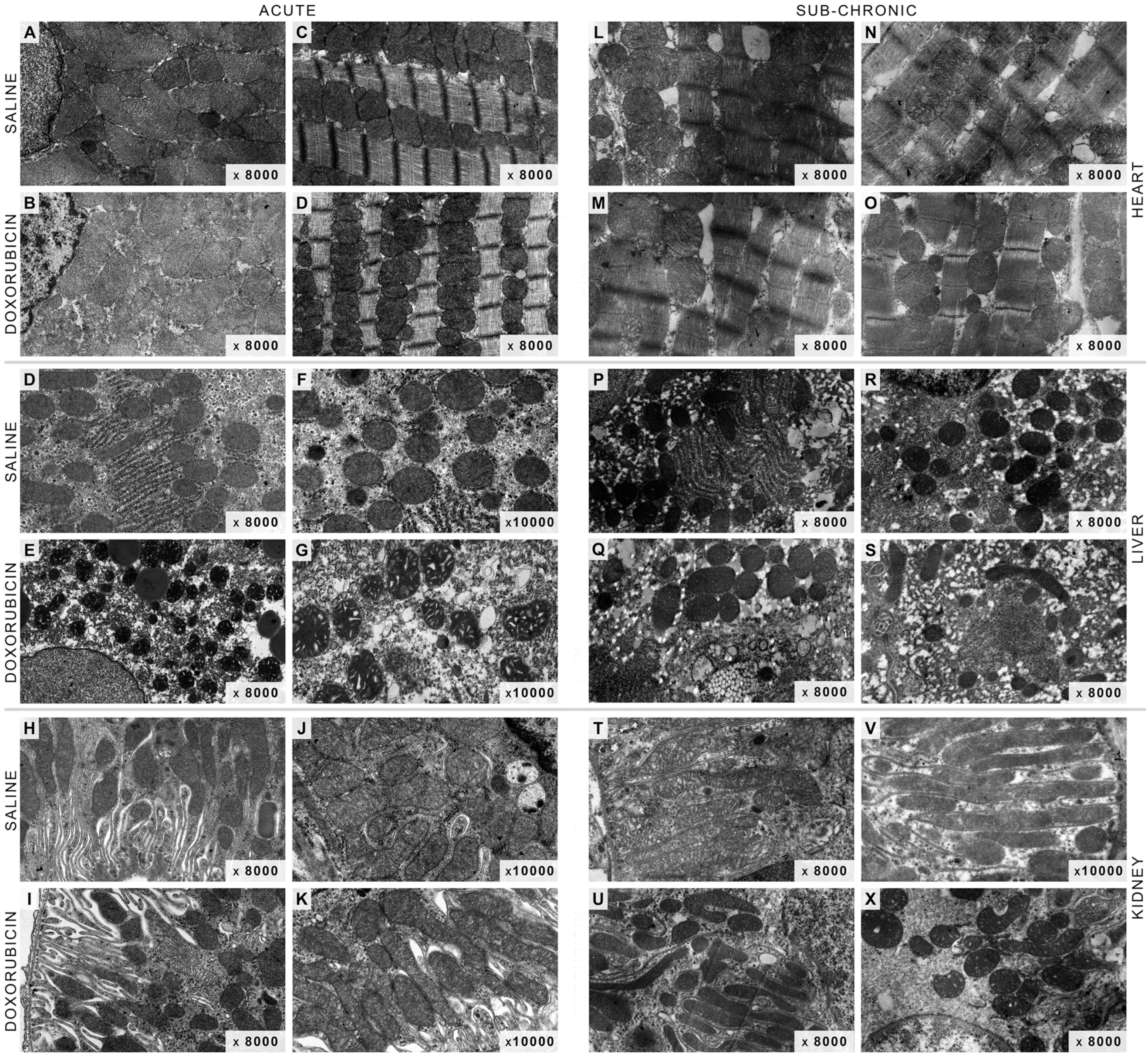

Figure 3. Cellular ultra-structure remains intact after acute or sub-chronic DOX treatment. No notorious differences or hallmarks of DOX toxicity were found in the different tissues in both protocols. Panels represent electron microphotographs of randomly chosen tissues: hearts present well-defined Z-bands and organized myofibrils (Panel **C**,**D** and **L**–**O**) and minor cytoplasmatic dilatation (Panel **M** and **O**); livers show cytoplasmatic vacuolization (Panel **E**, **G** and **S**) and lipid-like droplets structures (Panel **Q**) and some mitochondria appear in the condensed conformation (Panel **G**); renal mitochondria appear in an intermediated conformation between orthodox and condensed form (Panel **X**). Organs were fixed in 4% gluteraldehyde and post-fixed in osmium (for more information, see Material and Methods).
doi:10.1371/journal.pone.0038867.g003

dilated ventricles as a sign of hypertrophy, which was not observed in our two models.

The loss of cardiac structure and deteriorated function usually observed in long-term treatment with DOX [33,34] was not present in our model, supporting the idea of unaltered organ physiology. However, an interesting work performed on young mice demonstrated that chronic DOX treatment did not result in any sign of cardiomyopathy until animals were subject to a stressful swimming protocol [11]. The dissimilarities between studies, including our own, where no alterations in heart mass in DOX-treated rats were detected and other reports where those alterations were measured [8,11,35,36], may be explained by the different rat strains used or by the fact that the alterations may only be triggered in the presence of a physiological stress. In fact, this idea is put in use in the clinical practice where general diagnostic techniques for cardiac function, such as echocardiograms, are performed with increased workload and demands for higher cardiac output increasing therefore the specificity of screening and decreasing the number of false negatives [37,38].

In fact, the concept of normal organ physiology during resting conditions but altered when submitted to a stressful event led us to investigate mitochondrial function, since we can artificially

Table 4. Effects of DOX on mitochondrial transmembrane electric potential.

Model	Substrate	Treatment	$\Delta\Psi_{max}$ (-mV)						ADP Depolarization (mV)						Phosphorylative Lag Phase (sec)					
			Heart		Liver		Kidney		Heart		Liver		Kidney		Heart		Liver		Kidney	
			Mean	SE	Mean	SE	Mean	SE	Mean	SE	Mean	SE	Mean	SE	Mean	SE	Mean	SE	Mean	SE
Acute			n = 10		n = 9		n = 10		n = 10		n = 9		n = 10		n = 10		n = 9		n = 10	
	Glutamate Malate	Saline	222.2	3.9	226.3	5.7	219.2	2.6	33.7	2.5	27.7	2.6	27.3	1.4	30.6	5.5	51.5	6.6	22.8	1.8
		DOX	224.3	5.4	226.4	4.4	217.8	2.9	38.1	3.2	27.7	2.9	29.0	1.0	25.5	3.2	43.3	4.9	25.8	2.8
	Succinate	Saline	223.7	2.9	231.8	5.0	222.9	2.7	42.2	1.4	33.3	1.8	31.7	0.8	41.6	6.8	68.0	8.4	27.4	2.8
		DOX	227.7	2.2	227.3	2.0	223.1	2.9	44.8	1.4	32.6	1.4	32.2	1.3	48.6	10.4	64.0	10.5	27.3	2.9
Sub-Chronic			n = 12		n = 11		n = 12		n = 12		n = 11		n = 12		n = 12		n = 11		n = 12	
	Glutamate Malate	Saline	210.4	2.6	211.3	1.7	208.4	2.0	24.3	1.3	21.2	1.8	18.1	1.5	29.1	2.5	55.8	6.1	34.9	3.7
		DOX	208.1	3.1	207.4**	2.1	205.0**	1.7	24.2	0.9	22.4	1.4	18.9	1.9	32.2*	1.7	70.9*	11.5	38.9	3.5
	Succinate	Saline	214.9	2.6	217.0	1.6	211.1	3.4	27.0	1.6	26.2	1.7	19.7	2.0	38.4	3.1	64.4	6.2	38.2	3.6
		DOX	210.2**	3.0	216.5	2.4	209.2	4.1	27.0	1.7	26.5	1.9	19.2	2.2	43.5*	3.7	76.1	8.1	46.4	6.3

Data was collected with a TPP^+-sensitive electrode (for details see Materials and Methods) where 225–250 nmol ADP were added to induce depolarization (phosphorylative cycle). Differences between treatment groups means within the same model were evaluated by matched pairs Student's t test to exclude the variability related to mitochondrial isolation and electrode calibration but when assumptions were rejected the non-parametric Wilcoxon matched pairs test was applied (see Material and Methods for detailed information).

*$p \leq 0.05$;

**$p \leq 0.01$ vs saline group of the same model. SE – standard error.

doi:10.1371/journal.pone.0038867.t004

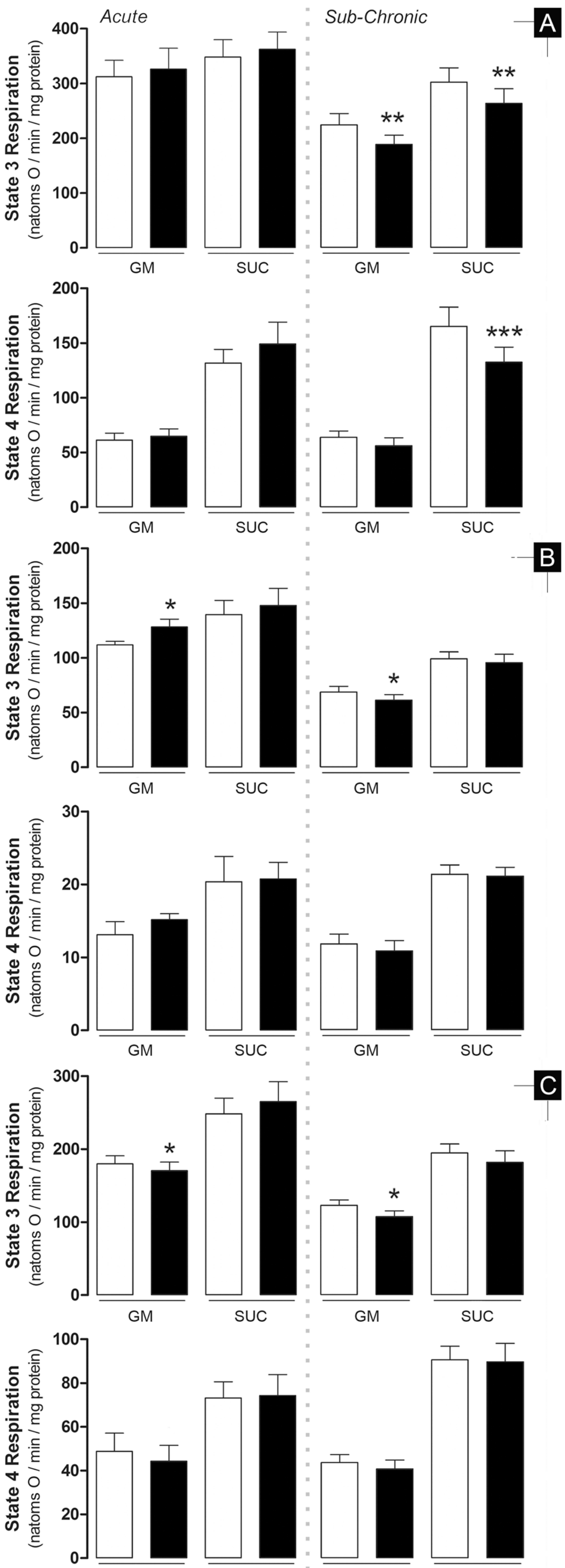

Figure 4. Treatment with DOX markedly affects mitochondrial respiration during ADP phosphorylation. The effect is clearly observed in heart mitochondria during sub-chronic treatment but absent when animals were acutely-treated. A reverse pattern is observed in the other two organs tested. Data represents mitochondrial oxygen consumption rates measured with a Clark electrode (for details, see Materials and Methods) where 225–250 nmol ADP was added to induce state 3 respiration. State 4 is generally described as the rate of oxygen consumption after complete phosphorylation of added ADP. **A** – heart; **B** – liver; **C** – kidney. Bars represent means of treatment groups (saline in **white bars**; DOX in **black bars**) with SE. Differences between treatment groups means within the same model were evaluated by matched pairs Student's t test to exclude the variability related to mitochondrial isolation and electrode calibration but when assumptions were rejected the non-parametric Wilcoxon matched pairs test was applied (see Material and Methods for detailed information). *, $p \leq 0.05$; **, $p \leq 0.01$; ***, $p \leq 0.001$ vs saline group of the same model. n = 10, 9 and 10 (acute model – heart, liver and kidney, respectively) or n = 12, 11 and 12 (sub-chronic model – heart, liver and kidney, respectively). GM - glutamate/malate; SUC - succinate.
doi:10.1371/journal.pone.0038867.g004

stimulate this model system, by creating a pseudo-metabolic stress by the addition of ADP. Furthermore, alterations of cardiac mitochondrial function were already described [37]. Once again, a different response to DOX treatment was observed in the two models. Mitochondrial alterations, as assessed by oxygen consumption and transmembrane electric potential, were noticeable in both treatment protocols but the degree of effect and their targets were distinct. If one assumes that the extension of statistical significance of the 7 distinct end-points regarding respiration/transmembrane electric potential (Table 4, Fig. 4 and 5) can indicate the extension of treatment damage to mitochondria (total of 14 parameters for tissue specificity, 21 for respiratory complex specificity and 42 for model specificity) we can make the following assumptions: a) Mitochondrial dysfunction is clearly more present in the sub-chronic model with 11/42 parameters altered in comparison to 2/42 in the acute, b) Cardiac mitochondria in the acute model are the less affected population with 0/14 altered parameters while both hepatic and renal mitochondria have at least one (1/14) altered parameter, c) Contrarily, heart mitochondria is the most affected group in the sub-chronic model with 6/14 altered parameters compared to the liver (3/14) and kidney (2/14) and d) DOX-treatment also leads to more alterations in complex-I sustained respiration with 2/21 and 8/21 altered parameters in acute and sub-chronic model, respectively, in comparison to complex-II sustained respiration which had 0/21 and 4/21 altered parameters in same treatment schedules, respectively. It seems therefore plausible to consider that mitochondrial bioenergetic dysfunction, together with harmful effects of DOX on energy substrate channeling, synthesis and availability [2,39] may be prior and thus responsible for altered cardiac metabolism and structure remodeling.

Oxygen consumption and calcium-loading capacity were previously reported to be accurate markers for DOX-induced mitochondriopathy [13,26,29,36,39]. Although some argue about the sensitivity of mitochondrial bioenergetics parameters [15], state 3 respiration was a good indicator for mitochondrial dysfunction in this study since it uncovered differences between tissues and treatment protocols. Importantly, changes in this parameter were detected before changes in organ structure or function occurred.

DOX is known for its futile redox cycle on mitochondrial complex I [40]. DOX enhances the production of ROS which is closely related to mitochondrial toxicity and further damage to cell tissue. In the present study, activity of the tricarboxylic acid cycle

Table 5. Effects of DOX on mitochondrial aconitase activity.

Model	Treatment	Heart		Liver		Kidney	
		Mean	SE	Mean	SE	Mean	SE
		%					
Acute	Saline (n = 6)	100	13.1	100	12.5	100	12.7
	DOX (n = 6)	100.3	12.8	85.0	18.9	72.2**	9.0
Sub-Chronic	Saline (n = 5)	100	10.4	100	14.7	100	11.7
	DOX (n = 5)	78.5*	14.5	112.9	25.9	105.4	12.4

Differences between treatment groups means within the same model were evaluated by matched pairs Student's t test to exclude the variability related to mitochondrial isolation (see Material and Methods for detailed information).
*p≤0.05;
**p≤0.01 vs saline group of the same model. SE – standard error.
doi:10.1371/journal.pone.0038867.t005

enzyme aconitase, an indirect but specific marker of oxidative stress, was used. The profile of enzyme inhibition followed the previous idea that DOX toxicity is treatment schedule and target-specific; cardiac aconitase activity was decreased in the sub-chronic model while it was unchanged in the acute (Table 5). The results suggest increased cardiac mitochondrial oxidative stress in the sub-chronic model but not in the acute; in this latter case, only kidney mitochondria presented decreased aconitase activity. This was rather surprising since we were expecting effects in both models due to the fact that DOX accumulates rapidly in the tissue and remains at high levels even after the treatment is ended [41]. In fact, DOX-induced increase in oxidative stress is exacerbated in long-term treatments since the primary damage of reactive oxygen species (ROS) on mitochondrial DNA can lead to a defective respiratory chain, increasing therefore the productions of ROS. Nevertheless, we believe that oxidative stress is indeed present in the acute model but perhaps antioxidant enzymes were also up-regulated, as previously described [42], although more work is needed in this regard. Interestingly, most published works done by using animal models and a single injection with higher DOX concentrations found evidences of cardiomyopathy several days later [14,43,44,45]. Although this observation is not consistent with the well-known late-onset DOX-induced cardiomyopathy, we believe that previous studies combined with our present data suggest a possible time window where strategies to counteract the drug toxicity can be effectively applied.

To our knowledge this was the first time that a 7+1 week treatment protocol was used in Wistar rats. Since different rat strains differ in their metabolism and susceptibility to toxic agents [46,47], data interpretation from the present work is not directly comparable to previous data using other rat strains, including Sprague-Dawley [8,15,26,35,36,48], which may present different tolerance to DOX. Interestingly, the results from Sprague-Dawley [6,9,27] and Wistar rats (our study) suggest that the former are more susceptible to DOX cardiotoxicity. Although needed to be confirmed by a new study, the differences between rat strains corroborate the idea of polymorphism-driven susceptibility to chemotherapy [49,50]. In fact, the same sub-chronic protocol in Sprague-Dawley caused extensive ascites (Oliveira, personal communication), a marker of heart failure, which was absent in the present work.

Our data confirms once again the idea of a preferential toxicity targeted to the heart although this was now clearly demonstrated in a multi-organ experimental model. Moreover, mitochondrial

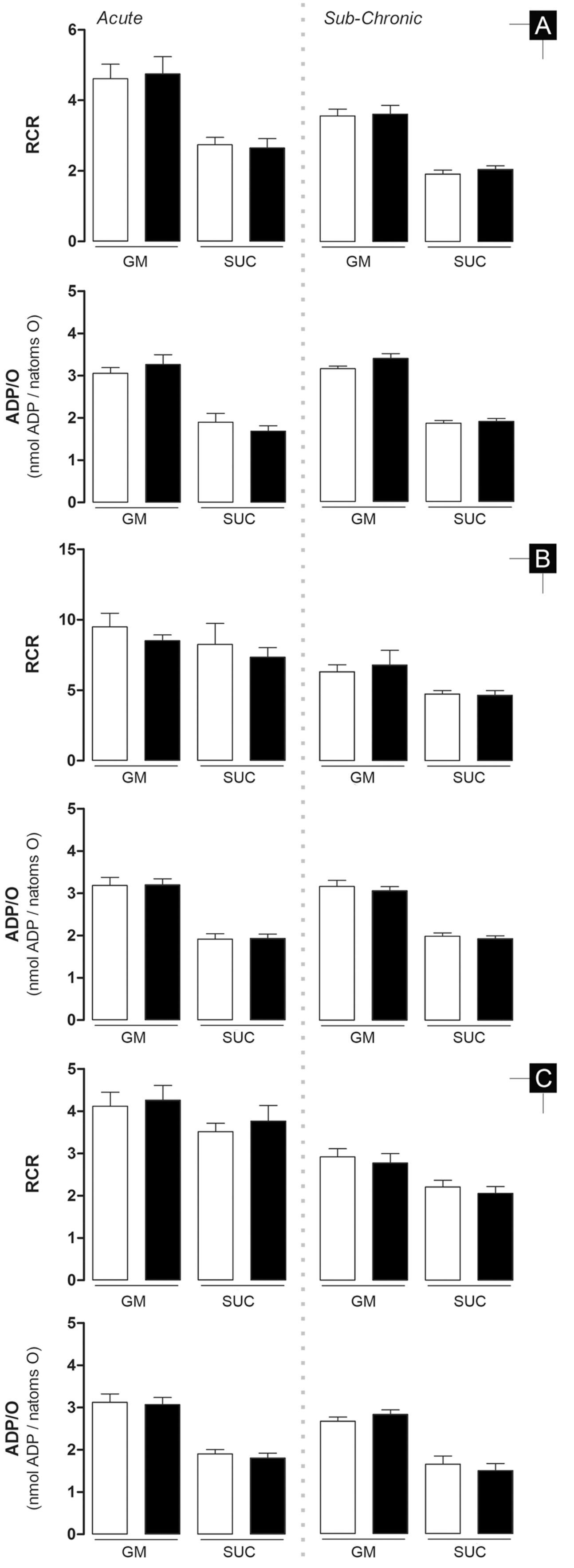

Figure 5. Respiratory Control Ratio (RCR) and ADP phosphorylated per consumed oxygen ratio (ADP/O). A – heart; **B** – liver; **C** – kidney. Bars represent means of treatment groups (saline in **white bars**; DOX in **black bars**) with SE. Differences between treatment groups means within the same model were evaluated by matched pairs Student's t test to exclude the variability related to mitochondrial isolation and electrode calibration but when assumptions were rejected the non-parametric Wilcoxon matched pairs test was applied (see Material and Methods for detailed information). *, $p \leq 0.05$ vs saline group of the same model. n = 10, 9 and 10 (acute model – heart, liver and kidney, respectively) or n = 12, 11 and 12 (sub-chronic model – heart, liver and kidney, respectively). GM - glutamate/malate; SUC - succinate.
doi:10.1371/journal.pone.0038867.g005

dysfunction is detected before detection of cardiomyopathy as assessed by echocardiography, or morphological changes. Animals appear to be mostly normal although presenting impaired cardiac mitochondrial function, which may pre-dispose these organelles for failure during stressful events. The results suggest that DOX cardiotoxicity is better revealed when animals models or humans are placed under stress, as referred in this study [11]. Stressful events can include pregnancy, which has been described to present a higher risk in survivors of childhood leukemia treated with DOX [51].

In conclusion, our data confirms that mitochondrial dysfunction is one major cause of DOX-selective cardiotoxicity and not a consequence as sometimes is questioned [29,30]. The present work is also the first to provide a three organ analysis of DOX toxicity using two different experimental protocols in Wistar rats. DOX did not cause substantial morphological or echocardiographic alterations in the heart or any other organs analyzed, although cardiac mitochondria showed alterations. Therefore, data confirms that mitochondrial alterations result from DOX treatment, being more severe in the heart and which are dependent on the treatment protocol. Thus, mitochondrial dysfunction is an early marker of DOX toxicity, although it remains to be determined if mitochondrial alterations in organs such as liver and kidney are a direct effect of DOX on mitochondria or instead if they result from secondary effects of DOX on other target tissues.

Supporting Information

Figure S1 PubMed results distribution of research involving "doxorubicin" and "mitochondria" according to tissue category. The Venn diagram presented in the figure was elaborated after collecting data from the PubMed website (assessment date February 27th) using specific *#keywords* to obtain the desired output. Briefly, papers in the database that included works related to the *#drug* and *#mitochondria* were retrived, restricting the output for research performed in the defined *#tissue*, excluding *#reviews* and works performed in *#humans* as long as they are not indexed with other animals. Therefore, the base of the search string was as follow: (((#mitochondria AND #drug) AND #tissue) NOT #reviews) NOT #humans Further explanation about each of the keywords is given in supporting Table 1. The authors recognize that the present search string is not flawless; however, the idea is to give the reader an overview of report rankings across the selected tissues. In fact, we acknowledge the fact that, for example, the keyword #humans will not include recent reports since they are yet to be indexed to Medline.
(TIF)

Table S1 Description of keywords used in PubMed search for construction of Venn diagram of Fig.S1, as well as the number of results retrieved with for each corresponding keyword.
(DOCX)

Acknowledgments

We are thankful to Dr. Lina Carvalho (Services of Pathological Anatomy and Medical Oncology, Hospitals of University of Coimbra) for histological analyses, Dr. Cristina Brás (Institute of Experimental Pathology, Faculty of Medicine, University of Coimbra) for echocardiograms and Dr. Mário Grãos (Biocant, Cantanhede, Coimbra) for the troponin I multiplex assay.

Author Contributions

Conceived and designed the experiments: GCP PJO AJM MSS. Performed the experiments: GCP SPP CVP JAL JM AA. Analyzed the data: GCP SPP AJM PJO JM AA JAL. Contributed reagents/materials/analysis tools: MSS AJM. Wrote the paper: GCP PJO.

References

1. Weiss RB (1992) The anthracyclines: will we ever find a better doxorubicin? Semin Oncol 19: 670–686.
2. Pereira GC, Silva AM, Diogo CV, Carvalho FS, Monteiro P, et al. (2011) Drug-induced cardiac mitochondrial toxicity and protection: from doxorubicin to carvedilol. Curr Pharm Des 17: 2113–2129.
3. Von Hoff DD, Layard MW, Basa P, Davis HL Jr, Von Hoff AL, et al. (1979) Risk factors for doxorubicin-induced congestive heart failure. Ann Intern Med 91: 710–717.
4. Steinherz LJ, Steinherz PG, Tan CT, Heller G, Murphy ML (1991) Cardiac toxicity 4 to 20 years after completing anthracycline therapy. JAMA 266: 1672–1677.
5. Wallace KB (2003) Doxorubicin-induced cardiac mitochondrionopathy. Pharmacol Toxicol 93: 105–115.
6. Oliveira PJ, Santos MS, Wallace KB (2006) Doxorubicin-induced thiol-dependent alteration of cardiac mitochondrial permeability transition and respiration. Biochemistry (Mosc) 71: 194–199.
7. Cardoso S, Santos RX, Carvalho C, Correia S, Pereira GC, et al. (2008) Doxorubicin increases the susceptibility of brain mitochondria to Ca(2+)-induced permeability transition and oxidative damage. Free Radic Biol Med 45: 1395–1402.
8. Berthiaume JM, Wallace KB (2007) Persistent alterations to the gene expression profile of the heart subsequent to chronic Doxorubicin treatment. Cardiovasc Toxicol 7: 178–191.
9. Zhou S, Starkov A, Froberg MK, Leino RL, Wallace KB (2001) Cumulative and irreversible cardiac mitochondrial dysfunction induced by doxorubicin. Cancer Res 61: 771–777.
10. Sacco G, Bigioni M, Evangelista S, Goso C, Manzini S, et al. (2001) Cardioprotective effects of zofenopril, a new angiotensin-converting enzyme inhibitor, on doxorubicin-induced cardiotoxicity in the rat. Eur J Pharmacol 414: 71–78.
11. Huang C, Zhang X, Ramil JM, Rikka S, Kim L, et al. (2010) Juvenile exposure to anthracyclines impairs cardiac progenitor cell function and vascularization resulting in greater susceptibility to stress-induced myocardial injury in adult mice. Circulation 121: 675–683.
12. Ahmed HH, Mannaa F, Elmegeed GA, Doss SH (2005) Cardioprotective activity of melatonin and its novel synthesized derivatives on doxorubicin-induced cardiotoxicity. Bioorg Med Chem 13: 1847–1857.
13. Ascensão A, Magalhães J, Soares JMC, Ferreira RM, Neuparth MJ, et al. (2005) Moderate endurance training prevents doxorubicin-induced in vivo mitochondriopathy and reduces the development of cardiac apoptosis. Am J Physiol Heart Circ Physiol 289: H722–731.
14. van Leeuwen BL, Kamps WA, Hartel RM, Veth RP, Sluiter WJ, et al. (2000) Effect of single chemotherapeutic agents on the growing skeleton of the rat. Ann Oncol 11: 1121–1126.
15. Oliveira PJ, Bjork Ja, Santos MS, Leino RL, Froberg MK, et al. (2004) Carvedilol-mediated antioxidant protection against doxorubicin-induced cardiac mitochondrial toxicity. Toxicol Appl Pharmacol 200: 159–168.
16. Takemura G, Fujiwara H (2007) Doxorubicin-induced cardiomyopathy from the cardiotoxic mechanisms to management. Prog Cardiovasc Dis 49: 330–352.
17. Brás C, Roque H, de Souza IA (2007) Echocardiographic Evaluation in Experimental Pathology. Exp Pathol Health Sci 1: 17–20.
18. Emanuelov AK, Shainberg A, Chepurko Y, Kaplan D, Sagie A, et al. (2010) Adenosine A3 receptor-mediated cardioprotection against doxorubicin-induced mitochondrial damage. Biochem Pharmacol 79: 180–187.
19. Oliveira PJ, Santos DJ, Moreno AJ (2000) Carvedilol inhibits the exogenous NADH dehydrogenase in rat heart mitochondria. Arch Biochem Biophys 374: 279–285.
20. Pereira SP, Fernandes MAS, Martins JD, Santos MS, Moreno AJM, et al. (2009) Toxicity assessment of the herbicide metolachlor comparative effects on bacterial and mitochondrial model systems. Toxicol In Vitro 23: 1585–1590.

21. Oliveira PJ, Esteves TC, Seiça R, Moreno AJM, Santos MS (2004) Calcium-dependent mitochondrial permeability transition is augmented in the kidney of Goto-Kakizaki diabetic rat. Diabetes Metab Res Rev 20: 131–136.
22. Gornal AC, Bardawill CJ, David MM (1949) Determination of serum proteins by means of the biuret reaction. J Biol Chem 177: 751–766.
23. Kamo N, Muratsugu M, Hongoh R, Kobatake Y (1979) Membrane potential of mitochondria measured with an electrode sensitive to tetraphenyl phosphonium and relationship between proton electrochemical potential and phosphorylation potential in steady state. J Membr Biol 49: 105–121.
24. Hausladen A, Fridovich I (1996) Measuring nitric oxide and superoxide: rate constants for aconitase reactivity. Methods Enzymol 269: 37–41.
25. Zar JH (1999) Biostatistical analysis: Prentice Hall.
26. Berthiaume JM, Oliveira PJ, Fariss MW, Wallace KB (2005) Dietary vitamin E decreases doxorubicin-induced oxidative stress without preventing mitochondrial dysfunction. Cardiovasc Toxicol 5: 257–267.
27. Oliveira PJ, Bjork JA, Santos MS, Leino RL, Froberg MK, et al. (2004) Carvedilol-mediated antioxidant protection against doxorubicin-induced cardiac mitochondrial toxicity. Toxicol Appl Pharmacol 200: 159–168.
28. Carvalho C, Santos RX, Cardoso S, Correia S, Oliveira PJ, et al. (2009) Doxorubicin: the good, the bad and the ugly effect. Curr Med Chem 16: 3267–3285.
29. Pereira GC, Oliveira PJ (2008) Pharmacological strategies to counteract doxorubicin-induced cardiotoxicity : the role of mitochondria. J Theor Exp Pharm 1: 39–53.
30. Minotti G, Menna P, Salvatorelli E, Cairo G, Gianni L (2004) Anthracyclines: molecular advances and pharmacologic developments in antitumor activity and cardiotoxicity. Pharmacol Rev 56: 185–229.
31. Sardao VA, Oliveira PJ, Holy J, Oliveira CR, Wallace KB (2009) Morphological alterations induced by doxorubicin on H9c2 myoblasts: nuclear, mitochondrial, and cytoskeletal targets. Cell Biol Toxicol 25: 227–243.
32. Lushnikova EL, Klinnikova MG, Molodykh OP, Nepomnyashchikh LM (2004) Morphological manifestations of heart remodeling in anthracycline-induced dilated cardiomyopathy. Bull Exp Biol Med 138: 607–612.
33. Migrino RQ, Aggarwal D, Konorev E, Brahmbhatt T, Bright M, et al. (2008) Early detection of doxorubicin cardiomyopathy using two-dimensional strain echocardiography. Ultrasound Med Biol 34: 208–214.
34. Shakir D (2009) Chemotherapy Induced Cardiomyopathy: Pathogenesis, Monitoring and Management. J Clin Med Res 1: 8–12.
35. Solem LE, Heller LJ, Wallace KB (1996) Dose-dependent increase in sensitivity to calcium-induced mitochondrial dysfunction and cardiomyocyte cell injury by doxorubicin. J Mol Cell Cardiol 28: 1023–1032.
36. Oliveira PJ, Wallace KB (2006) Depletion of adenine nucleotide translocator protein in heart mitochondria from doxorubicin-treated rats–relevance for mitochondrial dysfunction. Toxicology 220: 160–168.
37. Neilan TG, Jassal DS, Perez-Sanz TM, Raher MJ, Pradhan AD, et al. (2006) Tissue Doppler imaging predicts left ventricular dysfunction and mortality in a murine model of cardiac injury. Eur Heart J 27: 1868–1875.
38. Smibert E, Carlin JB, Vidmar S, Wilkinson LC, Newton M, et al. (2004) Exercise echocardiography reflects cumulative anthracycline exposure during childhood. Pediatr Blood Cancer 42: 556–562.
39. Tokarska-Schlattner M, Zaugg M, Zuppinger C, Wallimann T, Schlattner U (2006) New insights into doxorubicin-induced cardiotoxicity: the critical role of cellular energetics. J Mol Cell Cardiol 41: 389–405.
40. Doroshow JH (1983) Effect of anthracycline antibiotics on oxygen radical formation in rat heart. Cancer Res 43: 460–472.
41. Peters JH, Gordon GR, Kashiwase D, Acton EM (1981) Tissue distribution of doxorubicin and doxorubicinol in rats receiving multiple doses of doxorubicin. Cancer Chemother Pharmacol 7: 65–69.
42. Ascensao A, Lumini-Oliveira J, Machado NG, Ferreira RM, Goncalves IO, et al. (2011) Acute exercise protects against calcium-induced cardiac mitochondrial permeability transition pore opening in doxorubicin-treated rats. Clin Sci (Lond) 120: 37–49.
43. Riad A, Bien S, Westermann D, Becher PM, Loya K, et al. (2009) Pretreatment with statin attenuates the cardiotoxicity of Doxorubicin in mice. Cancer Res 69: 695–699.
44. Bai P, Mabley JG, Liaudet L, Virag L, Szabo C, et al. (2004) Matrix metalloproteinase activation is an early event in doxorubicin-induced cardiotoxicity. Oncol Rep 11: 505–508.
45. Todorova VK, Kaufmann Y, Hennings L, Klimberg VS (2010) Oral glutamine protects against acute doxorubicin-induced cardiotoxicity of tumor-bearing rats. J Nutr 140: 44–48.
46. Mas M, Sabater E, Olaso MJ, Horga JF, Faura CC (2000) Genetic variability in morphine sensitivity and tolerance between different strains of rats. Brain Res 866: 109–115.
47. Kacew S, Festing MF (1996) Role of rat strain in the differential sensitivity to pharmaceutical agents and naturally occurring substances. J Toxicol Environ Health A 47: 1–30.
48. Palmeira CMM, Serrano J, Kuehl DW, Wallace KB (1997) Preferential oxidation of cardiac mitochondrial DNA following acute intoxication with doxorubicin. Biochim Biophys Acta 1321: 101–106.
49. Deng S, Wojnowski L (2007) Genotyping the risk of anthracycline-induced cardiotoxicity. Cardiovasc Toxicol 7: 129–134.
50. Robert J, Morvan VL, Smith D, Pourquier P, Bonnet J (2005) Predicting drug response and toxicity based on gene polymorphisms. Crit Rev Oncol Hematol 54: 171–196.
51. Schwartz CL (1999) Long-term survivors of childhood cancer: the late effects of therapy. Oncologist 4: 45–54.
